# A multidimensional metabolomics workflow to image biodistribution and evaluate pharmacodynamics in adult zebrafish

**DOI:** 10.1242/dmm.049550

**Published:** 2022-08-16

**Authors:** Madelyn M. Jackstadt, Casey A. Chamberlain, Steven R. Doonan, Leah P. Shriver, Gary J. Patti

**Affiliations:** 1 Department of Chemistry, Washington University in St. Louis, St. Louis, MO 63130, USA; 2 Department of Medicine, Washington University in St. Louis, St. Louis, MO 63110, USA; 3 Center for Metabolomics and Isotope Tracing, Washington University in St. Louis, St. Louis, MO 63130, USA; 4 Siteman Cancer Center, Washington University in St. Louis, St. Louis, MO 63110, USA

**Keywords:** Zebrafish, Metabolomics, Pharmacodynamics, Mass spectrometry imaging, Drug discovery

## Abstract

An integrated evaluation of the tissue distribution and pharmacodynamic properties of a therapeutic is essential for successful translation to the clinic. To date, however, cost-effective methods to measure these parameters at the systems level in model organisms are lacking. Here, we introduce a multidimensional workflow to evaluate drug activity that combines mass spectrometry-based imaging, absolute drug quantitation across different biological matrices, *in vivo* isotope tracing and global metabolome analysis in the adult zebrafish. As a proof of concept, we quantitatively determined the whole-body distribution of the anti-rheumatic agent hydroxychloroquine sulfate (HCQ) and measured the systemic metabolic impacts of drug treatment. We found that HCQ distributed to most organs in the adult zebrafish 24 h after addition of the drug to water, with the highest accumulation of both the drug and its metabolites being in the liver, intestine and kidney. Interestingly, HCQ treatment induced organ-specific alterations in metabolism. In the brain, for example, HCQ uniquely elevated pyruvate carboxylase activity to support increased synthesis of the neuronal metabolite, N-acetylaspartate. Taken together, this work validates a multidimensional metabolomics platform for evaluating the mode of action of a drug and its potential off-target effects in the adult zebrafish.

This article has an associated First Person interview with the first author of the paper.

## Abstract (precis)

**Summary:** By using complementary metabolomics technologies, we describe a multidimensional workflow to image the biodistribution and measure the pharmacodynamics of a drug in the adult zebrafish.

## INTRODUCTION

The majority of therapeutics evaluated in preclinical testing fail, making drug development a high-risk and expensive process (Hay et al., 2014). Compounds that do advance to the clinic must accumulate at the sites of disease, engage their target, and demonstrate acceptable toxicity profiles. Commonly used models to evaluate the toxicity and efficacy of drug candidates in the preclinical phase include tissue-culture systems and mouse models. Of note, these systems have the drawbacks of failing to recapitulate complex physiology and potentially being cost prohibitive, respectively. An alternative model is the adult zebrafish, which shares approximately 70% gene homology with humans, is cost effective to maintain, can be bred in large numbers, and has a rapid maturation time (Spitsbergen and Kent, 2003; Howe et al., 2013; Truong et al., 2014; Cassar et al., 2020). An advantage of using zebrafish over other vertebrate model organisms is the simplicity of the route in which drugs can be introduced to study biodistribution and pharmacodynamics. Water-soluble chemicals can be added directly to the aquarium to enable uptake by the mouth, skin and gills (Dang et al., 2016). Although less frequently employed than embryos, adult zebrafish are of particular relevance to drug studies because individual organs can be dissected and subsequently evaluated by liquid chromatography/mass spectrometry (LC/MS) (Jia et al., 2018; Naser et al., 2021; Patton et al., 2021). Additionally, technologies to genetically modify adult animals, such as transgene electroporation in adult zebrafish (Callahan et al., 2018) and morpholino-mediated gene knockdown (Craig et al., 2010), are available for functional validation of drug targets.

Quantitative analysis of small molecules by mass spectrometry is broadly referred to as the field of metabolomics (Cajka and Fiehn, 2016). Depending upon the objectives of an experiment, metabolomics can be performed by using one or more complementary approaches. In untargeted metabolomics, researchers aim to profile small molecules of both endogenous and xenobiotic origin at the comprehensive scale. Although a global assessment of metabolism might reveal unexpected biochemical changes that provide mechanistic insight related to drug activity, untargeted metabolomics typically only reveals alterations in the relative levels of compounds. To determine the absolute concentration of a drug compound or metabolite, investigators generally rely on an experimental paradigm known as targeted metabolomics (Schwaiger-Haber et al., 2021). In these analyses, a selected set of reference chemicals are spiked into each biological matrix at known concentrations to correct for differences in ionization efficiencies. It is important to point out that both untargeted and targeted metabolomics have limitations for evaluating drug activity and biodistribution. First, most untargeted and targeted metabolomics experiments are label free and provide only a snapshot of metabolism. To capture pathway dynamics, isotopically labeled tracers must be introduced into the biological system and their transformation monitored (Zamboni et al., 2015). Second, the most commonly used technology for performing untargeted and targeted metabolomics is LC/MS, which is performed on homogenized tissues and, therefore, does not provide information about the anatomical location of compounds. To map the spatial distribution of small molecules, a metabolite-imaging approach is required, such as matrix-assisted laser desorption/ionization (MALDI) or desorption electrospray ionization (DESI) (Rubakhin et al., 2005; Eberlin et al., 2011).

In this study, we developed a multidimensional platform that integrates untargeted metabolomics, absolute drug quantitation, *in vivo* isotope tracing and mass spectrometry-based imaging to examine the biodistribution and pharmacodynamics of hydroxychloroquine sulfate (HCQ) in adult zebrafish. An advantage of using adult zebrafish for such an analysis is that isotope tracers can be easily administered to animals by simply adding them to the tank water. Additionally, the size of the adult zebrafish is uniquely suited for biodistribution studies because a cross-section of the entire animal can be readily analyzed by MALDI or DESI imaging in a single experiment at a resolution that enables visualization of metabolic organs. Originally developed as an antimalarial in the drug class of 4-aminoquinolines, HCQ has been prescribed for long-term use in rheumatic diseases, including rheumatoid arthritis and systemic lupus erythematosus (Browning, 2014; Schrezenmeier and Dörner, 2020). HCQ has pleiotropic actions *in vivo* that have complicated our understanding of its mechanism of action and optimal dosing (Fox, 1993; Nirk et al., 2020; Schrezenmeier and Dörner, 2020). Here, by imaging HCQ accumulation in animals, we show that differential uptake of the drug in organs contributes to unique alterations in metabolism. We further demonstrate that, despite accumulating at low concentrations in the brain, HCQ increases pyruvate carboxylase activity to support elevated synthesis of N-acetylaspartate, a marker of neuronal integrity (Moffett et al., 2007). Taken together, these results validate a multidimensional workflow in adult zebrafish to obtain quantitative information on drug distribution, in addition to systems-level profiling of drug activity and toxicity for efficient preclinical evaluation of drugs.

## RESULTS

### Monitoring drug uptake in adult zebrafish by LC/MS

**Fig. 1.**
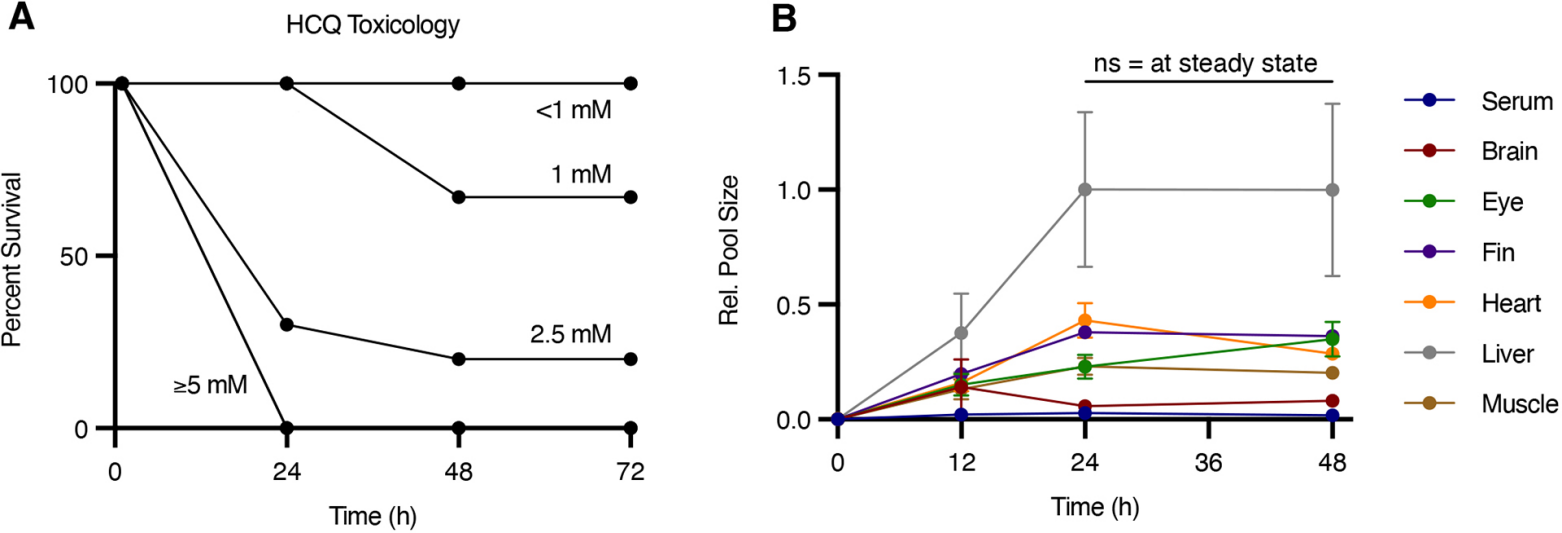
**Toxicity and biodistribution of HCQ in adult zebrafish.** (A) Toxicology profile for adult sjA zebrafish treated with HCQ. Survival is plotted as a function of exposure time (h); *n*=10 fish per group. (B) Accumulation of HCQ in serum and organs (relative to liver 24 h samples) after 1 mM HCQ treatment for 24 h. These data demonstrate steady-state conditions at 24 h. Values are mean±s.e.m.; *n*=3-4 fish per time point. For the 0 h time point, *n*=3 for liver, and *n*=4 for serum, brain, eye, fin, heart and muscle. For the 12 h time point, *n*=3 for serum, and *n*=4 for brain, eye, fin, heart, liver and muscle. For the 24 h time point, *n*=4 for all organs. For the 48 h time point, *n*=3 for serum, brain and heart, and *n*=4 for eye, fin, liver and muscle. Statistical significance was evaluated with a two-tailed, unpaired *t*-test; ns, not significant.

We first explored the toxicity profile of HCQ in wild-type (WT) adult zebrafish. Fish were placed in concentrations of HCQ that ranged from 1 µM to 10 mM and survival was monitored over time. Similar to embryos, adult zebrafish take up drugs dissolved in their tank water (Dang et al., 2016; Jia et al., 2018; Cassar et al., 2020). At concentrations of HCQ above 2.5 mM, we observed considerable mortality. When treating WT animals with 1 mM HCQ for 24 h, however, no animal death occurred (Fig. 1A). Using this concentration of 1 mM, we next monitored the kinetics of HCQ accumulation within the adult zebrafish. We placed fish in 1 mM HCQ for 12, 24 and 48 h, and then monitored the relative concentration of HCQ in various tissues compared to that in the liver, which is a well-characterized site of HCQ accumulation and metabolism (Schrezenmeier and Dörner, 2020). Steady-state drug concentrations were reached by 24 h for all tissues tested (Fig. 1B). These data indicate that this drug concentration and exposure time are appropriate for steady-state metabolomic studies.

### Mass spectrometry imaging of drug metabolism

LC/MS necessitates that samples be homogenized prior to analysis and, therefore, it does not provide information about tissue heterogeneity or the spatial distribution of chemicals. Unlike LC/MS, mass spectrometry-based imaging techniques such as DESI can be applied to intact sections of tissue. Accordingly, a single DESI imaging experiment can provide a map of drug distribution across the entire zebrafish animal. We applied DESI imaging to adult fish treated with 1.5 mM of HCQ for 24 h to determine the sites of drug accumulation and metabolism. Untreated animals were used as controls to assess potential background signals. After euthanizing the fish and amputating their caudal fin, the animals were exsanguinated to remove interference from HCQ and its metabolites in blood prior to imaging.

**Fig. 2.**
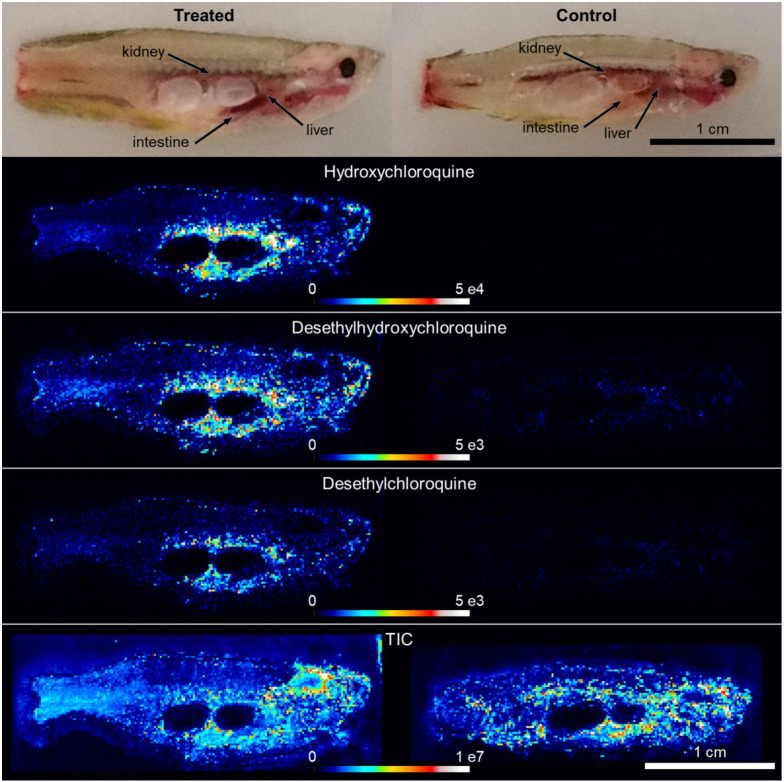
**DESI-MS imaging localizes HCQ and its metabolites to specific organs in zebrafish.** Annotated reference photographs (top) for collected sections of representative HCQ-treated (1.5 mM, 24 h) and control (no treatment) zebrafish. HCQ and its metabolites, desethylhydroxychloroquine and desethylchloroquine, were detected in high abundance (compared to control tissue), with enrichment in the intestine, liver and kidney. Comparable intensity in total ion current (TIC, described in Materials and Methods) confirmed stable DESI performance. Figures are representative images from a single section of each fish. *n*=2 per group (see Fig. S1B for replicate images). Scale bars: 1 cm.

DESI imaging of drug-treated zebrafish showed localization of HCQ in three primary organs. The liver and intestine had high uptake of HCQ and accumulated its primary metabolites, desethylhydroxychloroquine and desethylchloroquine (Fig. 2; Fig. S1A,B). This result is in line with the degradation of 4-aminoquinolines by the cytochrome P450 enzymes, CYP2C8 and CYP3A4, both present in the gastrointestinal tract and liver in humans (Dresser et al., 2000; Enayetallah et al., 2004). Liver and intestinal tissues from adult zebrafish are known to express *cyp3a65*, which has 54% similarity to human *CYP3A4* (Tseng et al., 2005; Goldstone et al., 2010). The kidney was the third organ that showed high signals from HCQ and related metabolites, indicating that renal clearance as a mechanism for drug elimination is shared between fish and humans (Browning, 2014). It is notable that the third primary metabolite of HCQ, bidesethylchloroquine (or bisdesethylchloroquine), was not detected in any experiments (neither by DESI imaging nor by LC/MS), likely due to the relatively low rate of bioconversion (Brown et al., 1986; Browning, 2014). We point out that only low levels of HCQ were detected in the heart, demonstrating that not all centrally located tissues accumulated HCQ to the extent that the liver and intestine did. All fish analyzed by DESI imaging had metabolite signals across the entire body of the animal, but not in the cavity left by the swim bladder. This result was best visualized by the total ion current (TIC), which is the sum of all signals detected. As expected, metabolites were concentrated in major organs such as the brain and heart in addition to the liver, intestine and kidney. Additionally, when HCQ images were scaled to the TIC, the patterns of localization remained similar to non-normalized data (Fig. S1C), corroborating that HCQ accumulation in the intestine, liver and kidney was not due to signal intensity fluctuations across the fish. These data provide confirmation that the DESI imaging platform is well suited for the measurement of exogenous drugs as well as endogenous metabolites in the adult zebrafish.

### Absolute quantitation of drug across tissues using a matrix-matched calibration curve

The activity of a drug is dependent on both its accumulation in the appropriate organ and binding to the desired protein target (Ijzerman and Guo, 2019; An, 2020). Therefore, quantitative assessment of drug concentrations within an organism is needed to properly interpret drug efficacy. Although DESI imaging can reveal the relative levels of a given drug throughout the body of a zebrafish, measuring the absolute concentration of the chemical across different tissues is best accomplished by performing targeted metabolomics with LC/MS. Unlike DESI imaging, LC/MS does not require tissue sectioning and, therefore, avoids potential problems such as signal distortions around tissue edges. The general practice when using LC/MS for quantitation is to create a calibration curve for each biological matrix (Dahal et al., 2011; Loos et al., 2016). In our case, we spiked different concentrations of the drug (together with a labeled version of the drug at a single concentration as an internal standard) into metabolic extracts from each zebrafish organ. It is important that the organs were obtained from animals that had not been administered HCQ, so that the only source of the drug was from the spike in. The purpose of creating a calibration curve for organs was to account for differences in drug ionization that result due to the presence of unique biological matrices (Fig. S2A).

A major limitation of creating calibration curves for each organ in the zebrafish is the resources and time required. In this particular study in which we aimed to quantitate drug concentrations throughout the animal, it required doing experiments with seven sets of individual tissues. Altogether we evaluated five concentrations of the drug in triplicate for each tissue, amounting to 105 LC/MS injections (Table S1). As an alternative, we considered the possibility of using a single calibration curve from a pooled set of tissues to quantitate drug concentrations across the entire zebrafish. To that end, we mixed intestine, liver, heart, eye, muscle, fin and brain tissue from animals that had not been treated with the drug. We then spiked in the drug at different known concentrations to create a single calibration curve, which we subsequently used to quantitate drug concentrations in all tissues analyzed. Using a single calibration curve from pooled tissues reduced the number of LC/MS injections from 105 to 15, but the drug concentrations determined were not significantly different from when we used tissue-specific calibration curves (Fig. S2B). The linear equations for each method are provided in Table S1, all of which showed high correlation coefficients (r>0.998). Our results demonstrate that using pooled tissues to approximate the biological matrix for quantitation is an efficient and accurate way to streamline drug-biodistribution studies.

**Fig. 3.**
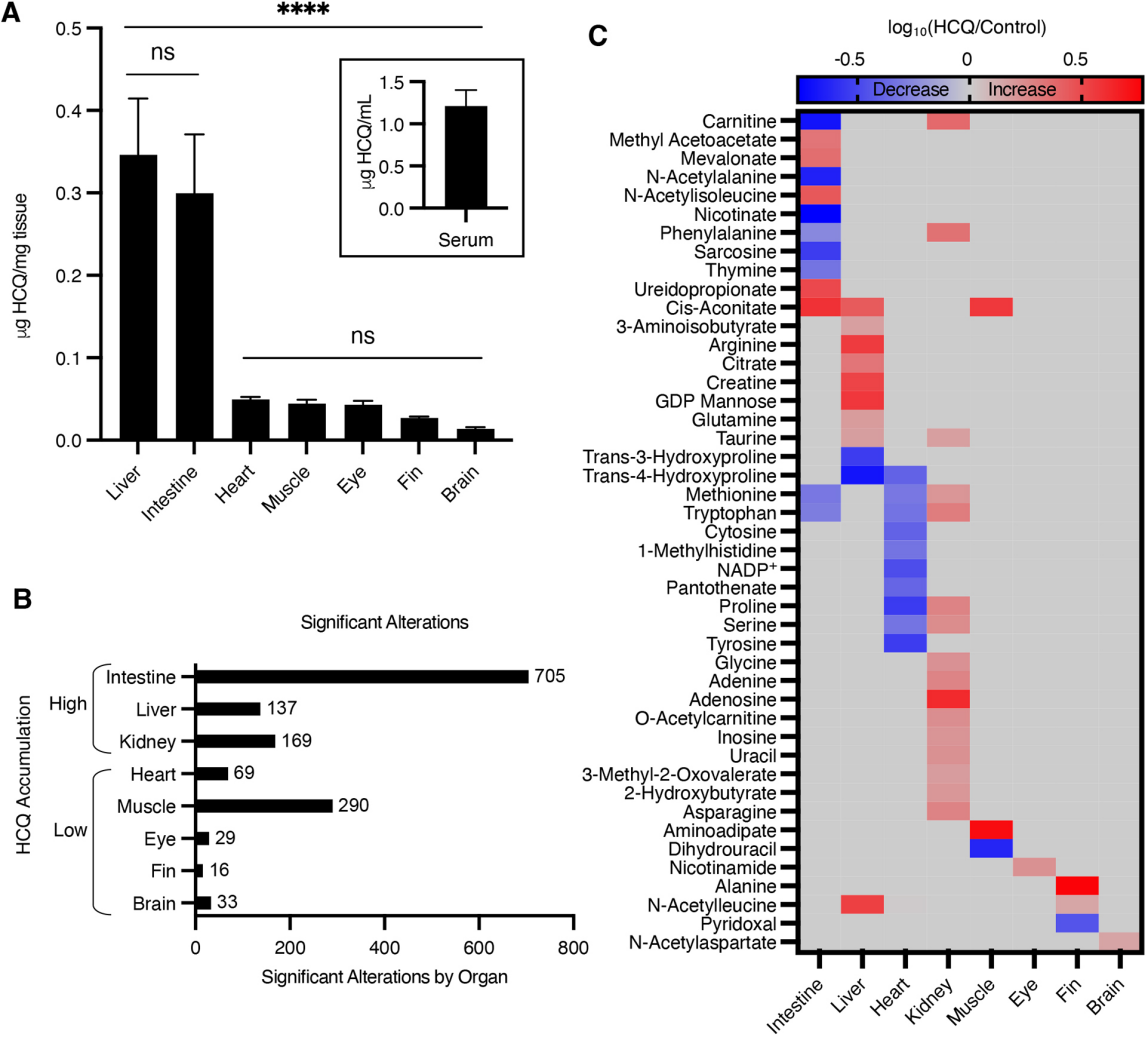
**Systems-level analysis of metabolic alterations reveals tissue-specific differences in metabolism after HCQ treatment.** (A) Absolute quantitation of hydroxychloroquine (HCQ) in organs (μg HCQ/mg tissue) and serum (μg HCQ/ml serum) of zebrafish treated with 1 mM HCQ for 24 h, showing high accumulation in the liver and intestine. Values are mean±s.e.m.; *n*=5-8 fish per group. For liver, *n*=5; for heart and intestine, *n*=7; for all other organs, *n*=8. (B) Histogram showing the count of statistically significant metabolic alterations in organs from HCQ-treated animals relative to untreated controls. Negative-ion mode data shown; *n*=5-8 fish per group. For control fish, *n*=5 for kidney and liver; *n*=6 for brain, eye, fin, heart, intestine and muscle. For HCQ-treated fish, *n*=7 for muscle and intestine; *n*=8 for kidney, brain, eye, fin, heart and liver. (C) Heatmap of select metabolites showing a statistically significant difference between HCQ-treated and control zebrafish. Increases in HCQ-treated animals are shown in red and decreases are shown in blue. Negative- and positive-mode data shown; *n*=5-8 fish. For control fish, *n*=5 for kidney and liver; *n=*6 for brain, eye, fin, heart, intestine and muscle. For HCQ-treated fish, *n*=7 for muscle and intestine; *n*=8 for kidney, brain, eye, fin, heart and liver. Statistical significance was evaluated with a one-way ANOVA with Tukey's method at 95% confidence (A) and with a two-tailed, unpaired *t*-test (C). ns, not significant; ****P<0.0001.

Absolute quantitation data from targeted LC/MS analyses corroborated the general localization patterns observed from DESI imaging (Fig. S2C). HCQ accumulated in the liver and intestine at more than three times its concentration in other organs (Fig. 3A), which is consistent with the known roles of the liver and intestine in metabolism (Pond and Tozer, 1984). These findings indicate that the distribution of HCQ is similar between zebrafish and humans, highlighting the utility of this organism as a model system for pharmacokinetic analysis (Costedoat-Chalumeau et al., 2020). Additionally, with LC/MS, we were able to quantitate the absolute concentration of HCQ in the serum of individual adult zebrafish. To collect a sufficient amount of material while minimizing perturbations to metabolism, we used low-speed centrifugation to collect blood from the amputated caudal fin of anesthetized animals. We have previously shown that this strategy for obtaining blood from adult zebrafish is more efficient than direct pipetting methods (Naser et al., 2021).

### Profiling organ-specific HCQ pharmacodynamics

We next sought to identify HCQ-related changes in metabolism that could be linked to either drug efficacy or toxicity. Organs from zebrafish exposed to 1 mM HCQ for 24 h were compared to organs from untreated animals by LC/MS-based untargeted metabolomics. We also performed LC/MS experiments on blank controls that did not contain any zebrafish specimens to annotate background ions originating from non-biological sources. Using the computational strategies that we established before, zebrafish datasets were processed to remove contaminants and degeneracies such as salt adducts (Mahieu and Patti, 2017; Cho et al., 2021). The signals that remained after filtering represented unique metabolites that are of physiological relevance. Surprisingly, the number of metabolites with statistically significant alterations in each organ did not correspond to the concentration of the drug in that tissue (Fig. 3B). The intestine, a site of considerable HCQ accumulation, showed the most metabolic changes. Muscle, on the other hand, had the second highest number of metabolite alterations despite its relatively low uptake of HCQ. Moreover, even though the liver accumulated the highest concentration of the drug, it had a comparatively small number of altered metabolites.

**Fig. 4.**
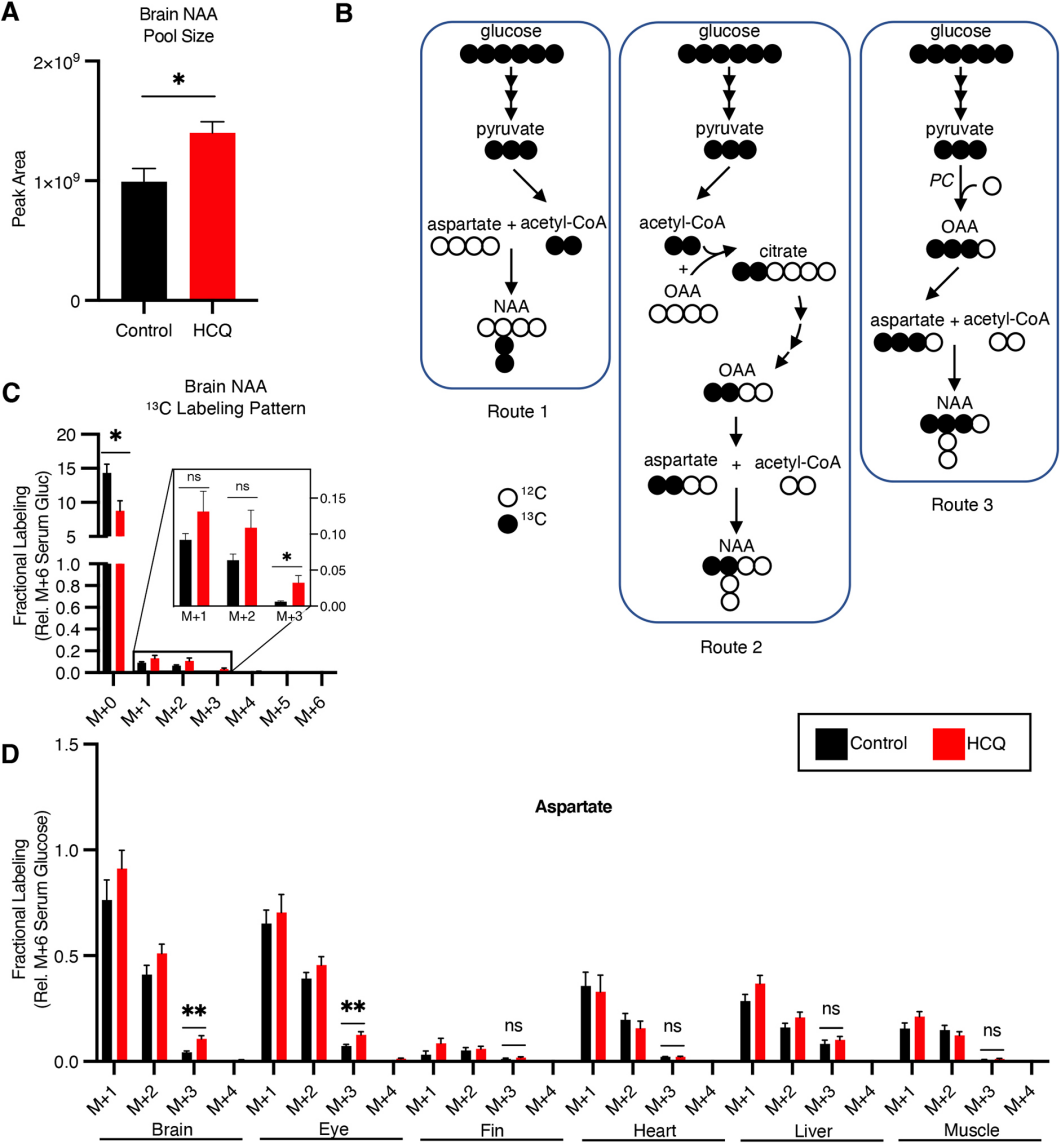
**HCQ increases NAA biosynthesis and pyruvate carboxylase activity in the brain.** (A) Pool size of NAA in the brain of HCQ-treated fish compared to untreated controls; *n*=6-8 fish per group. For control brains, *n*=6; for HCQ brains, *n*=8. (B) Labeling diagram to show three routes for glucose carbon to contribute to NAA biosynthesis. Pyruvate carboxylase, PC. (C) Fractional labeling (relative to serum M+6 glucose) of NAA in brain tissue of HCQ-treated zebrafish versus controls; *n*=6-9 fish per group. For control brains, *n*=6; for HCQ brains, *n*=9. (D) Fractional labeling of aspartate from ^13^C_6_-glucose in HCQ-treated and control zebrafish; *n*=6-9 fish per group. For control fish, *n=*6 for brain; *n*=8 for eye, fin, heart, liver and muscle. For HCQ-treated fish, *n*=8 for heart and liver; *n*=9 for brain, eye, fin and muscle. An expanded plot is shown in Fig. S3A. Statistical significance was evaluated with a two-tailed, unpaired *t*-test. ns, not significant; *P<0.05; **P<0.01.

Another interesting trend that we observed in the data is that the same metabolic alterations were not conserved across all tissues. To gain additional biochemical insight into organ-specific differences, we identified metabolites that only showed altered levels in particular tissues. All identifications were supported by matching experimental data from zebrafish to data obtained from using authentic reference standards, thereby providing level 1 confidence according to the guidelines suggested by the Metabolomics Standards Initiative (Sumner et al., 2007; Stancliffe et al., 2021). We found that HCQ treatment uniquely decreased the levels of some amino acids in the heart. In contrast, both the liver and kidney showed general increases in nitrogen-containing small molecules such as amino acids and nucleotides. In addition, the liver also showed alterations in the tricarboxylic acid (TCA) cycle intermediates citrate and cis-aconitate. One striking alteration that we only observed in the brain was related to N-acetylaspartate (NAA). Although the brain accumulated the lowest amount of HCQ, a statistically significant increase in NAA levels was measured (Fig. 3C, Fig. 4A). These data are consistent with organ-specific regulation of metabolism in response to drug treatment.

### HCQ increases pyruvate carboxylase activity in the brain

NAA is one of the most abundant small molecules in the brain. It is a general marker of neuronal health (Stovell et al., 2017), participates in neuron-oligodendrocyte communication (Singhal et al., 2017), and serves as a biosynthetic precursor to the important neurotransmitter N-acetylaspartylglutamate (NAAG) (Tsai and Coyle, 1995; Pouwels and Frahm, 1998; Moffett et al., 2007). We surmised that the increased levels of NAA that we observed in the brain of HCQ-treated fish might be supported by increased NAA biosynthesis. To test this prediction, we conducted an isotope-tracing experiment with ^13^C_6_-glucose. Adult zebrafish were randomly transferred to one of two aquariums containing 10 mM ^13^C_6_-glucose for 24 h. We already established in prior work that these conditions lead to steady-state labeling of glucose in the circulation of adult zebrafish and pseudo isotopic steady state in other tissues (Naser et al., 2021). In this work, in addition to 10 mM ^13^C_6_-glucose, one aquarium contained 1 mM HCQ. The other aquarium contained 10 mM ^13^C_6_-glucose but no HCQ as a control.

We used ^13^C_6_-glucose as a tracer because it can be transformed into both precursors of NAA, acetyl-CoA and aspartate (Moffett et al., 2007). Specifically, there are three routes by which the labeled carbon atoms can be incorporated into NAA from ^13^C_6_-glucose (Fig. 4B,C). In the first route, ^13^C_6_-glucose produces ^13^C_2_-acetyl-CoA, which is directly used to synthesize NAA. In the second route, glucose-derived ^13^C_2_-acetyl-CoA contributes its activated carbon to the TCA cycle, which subsequently produces labeled oxaloacetate. Oxaloacetate equilibrates with aspartate, which can be used to synthesize NAA with two labels (M+2, where M indicates the mass of the unlabeled metabolite). In the third route, ^13^C_6_-glucose is transformed into ^13^C_3_-pyruvate, which then contributes all three of its labeled carbons to oxaloacetate via pyruvate carboxylase activity. The third route produces NAA with three labels (M+3). We also observed NAA containing one label (M+1), which is synthesized from M+1 aspartate (Fig. 4D) and unlabeled acetyl-CoA. It has been suggested that when providing animals with ^13^C_6_-glucose as a tracer, M+1 aspartate might originate from malic enzyme activity (Sun et al., 2017).

By monitoring the pattern of label incorporation into NAA, we aimed to assess potential changes in NAA biosynthesis. Notably, neither M+1 nor M+2 NAA showed any statistically significant differences between HCQ-treated and control animals. We did find, however, that M+3 NAA was elevated in animals exposed to the drug (Fig. 4C). We reasoned that the increase in M+3 NAA was due to elevated pyruvate carboxylase activity, which provides a source of carbon for the TCA cycle to replace the oxaloacetate being removed for NAA biosynthesis. As additional evidence of elevated pyruvate carboxylase activity in the brain of drug-treated zebrafish, we found that the level of the M+3 isotopologue of aspartate and several other TCA cycle intermediates were increased (Fig. 4D; Fig. S3A,B). We point out that no other tissues showed similar labeling patterns indicative of elevated pyruvate carboxylase activity, except the eye (Fig. 4D; Fig. S3C). We conclude that the contribution that the third route provides to NAA is increased relative to the first and second routes in the brain and eye tissue of HCQ-treated fish.

## DISCUSSION

Most drug-discovery workflows using zebrafish are based on screening embryos, which have many attractive characteristics relative to other model organisms, as has been comprehensively reviewed previously (Scholz, 2013). When studying metabolism, however, the adult zebrafish has a key technical advantage over embryos in that its size enables metabolic analysis of organs and blood from individual animals by mass spectrometry (Naser et al., 2021). Similar to embryos, drugs can be administered to adult zebrafish by simply dissolving them in aquarium water. Subsequent metabolomics analysis then allows for the unique characterization of the pharmacokinetic and pharmacodynamic properties of a drug. Additionally, comprehensive metabolite profiles generated from each organ after drug exposure have the potential to provide new mechanistic insight into the mode of action and toxicity of the drug.

The main objective of the current work was to establish a multidimensional platform that exploits recent advances in metabolomics technologies to study drug activity at a comprehensive level in the adult zebrafish. We focused on combining four mass spectrometry-based approaches, providing highly complementary information that is critical in the process of drug discovery and development. We used HCQ as a proof of principle here, but the workflow we describe is broadly applicable to any compound that is water soluble. First, we mapped the distribution of HCQ across the entire zebrafish by using *in situ* metabolite imaging. The DESI imaging approach we applied has several important benefits. Unlike some other commonly used mass spectrometry-based imaging techniques, it does not require the addition of a matrix and, therefore, avoids background signals that potentially interfere with small-molecule drug detection (Guo et al., 2002; Patti et al., 2010). Second, by imaging longitudinal sections of animals, we were able to monitor drug accumulation in organs that are challenging to dissect for LC/MS analysis, such as the pancreas (Arjmand et al., 2020). In contrast to LC/MS experiments in which an entire organ is homogenized prior to analysis, DESI imaging also allows different functional areas of a tissue to be spatially resolved. The disadvantage of DESI imaging is that it only allows for semi-quantitative analysis of drug levels. To determine absolute drug concentrations, we integrated a targeted metabolomics approach that relies on creating a calibration curve with an authentic reference standard. Although it is general practice to create a calibration curve for each biological matrix evaluated, in this case, each organ, the process is time consuming and resource intensive. Thus, as an alternative, we explored the possibility of using one calibration curve from a set of pooled zebrafish tissues. We validated that this higher-throughput method did indeed have comparable quantitative accuracy relative to organ-specific calibrations. To complement analyses of drug levels, we applied a third experimental strategy known as untargeted metabolomics in which metabolites are profiled from individual zebrafish tissues at the global scale. Untargeted metabolomics can be applied not only to examine known mechanisms, but also to discover metabolic alterations associated with unexpected drug activities. Notwithstanding, untargeted metabolomics evaluates the relative differences in metabolite levels, which only provides a static snapshot of metabolism. To gain additional knowledge about the dynamics of metabolic pathways after drug treatment, we integrated a fourth technique into our workflow, which monitors the fate of an isotopically labeled tracer. Zebrafish are particularly well suited to study drug activity by using isotope-tracer analysis as the labeled nutrient can be added directly to tank water with the drug (Naser et al., 2021). Together, this combination of advanced metabolomics technologies provides an unprecedented level of resolution to study drug activity in adult zebrafish.

A unique characteristic of our platform is that we can measure metabolic processes from each major organ in a single set of experiments. For our proof-of-principle work, this led to the interesting observation that HCQ induces unique metabolic changes in different tissues. It is intriguing to consider why a drug might have organ-specific effects. One possibility is that the drug accumulates at different levels throughout the animal, thereby having varying degrees of activity and perhaps engaging unique targets at higher concentrations (Yao et al., 2020). Another possibility is that different tissues exhibit alternative degrees of metabolic flexibility (Galgani and Fernández-Verdejo, 2021). Some cell types might be highly sensitive to blocking a biochemical pathway, for example, whereas others might have compensatory mechanisms in place to overcome metabolic inhibition. It is also conceivable that the pathway targeted by a drug might not be active or required for all organs to function, or that chemical inhibition in one tissue changes physiological interactions between other tissues. To date, these sorts of chemical mechanisms have been challenging to study, owing to a paucity of techniques for comprehensively assessing how metabolism changes within each organ upon drug treatment. The workflow that we have established here for the adult zebrafish helps address this gap by simultaneously surveying metabolism at both the organ and systemic levels in a single set of experiments. In our proof-of-principle study, we found that HCQ and its major metabolites (desethylchloroquine and desethylhydroxychloroquine) were concentrated in the liver, intestine and kidney. Our findings indicate that these organs are the major sites of HCQ accumulation and metabolism in zebrafish, which is consistent with drug distribution data from mice (Chhonker et al., 2018), cynomolgus macaques (Liu et al., 2021) and humans (Van Eaton and Gustafson, 2021). Interestingly, the levels of HCQ accumulation did not correlate with the magnitude of metabolic changes. As an example, we observed a high level of metabolic dysregulation in the muscle, where HCQ levels were relatively low compared to those in the liver and intestine. Several mechanisms of action have been proposed for HCQ, including inhibition of autophagy and suppression of inflammatory cytokine release (Gies et al., 2020). Although the exact relationship between these processes and our metabolic changes remains to be defined, we observed several interesting organ-specific metabolic signatures in HCQ-treated zebrafish. The kidney showed increases in the renoprotective molecules acetylcarnitine and glycine (Wang et al., 2018), which might play a role in the mitigation of lupus-induced kidney injury by HCQ (Gómez-Guzmán et al., 2014). In contrast to its protective role in the kidney, HCQ can induce oxidative stress in muscle, leading to drug-induced myopathy (Uzar et al., 2012). We observed significant decreases in the levels of metabolites in muscle, including the redox-associated metabolites NADP^+^, serine and methionine. Moreover, although the brain had low amounts of HCQ, the brain and eye were unique in that they had increased pyruvate carboxylase activity. This metabolic adaptation might be related to distinct biochemical processes that are specific to the nervous system, such as production of neurotransmitters.

Predicting drug efficacy and toxicity in the preclinical stage remains one of the most challenging problems in drug development. Adult zebrafish have previously been used to screen for specific pathologies such as drug-induced kidney injury (Kato et al., 2020), inhibitors of fin regeneration (Oppedal and Goldsmith, 2010) and new chemotherapeutics (Nguyen et al., 2012). The workflow introduced here will augment this prior work that utilized phenotypic screens by providing a systems-level view of drug activity, therapeutic outcomes and toxicity at the metabolic level throughout the entire animal.

## MATERIALS AND METHODS

### Reagents

All solvents and reagents used for metabolite extraction and analysis by LC/MS and DESI imaging were of LC/MS grade. Acetonitrile, methanol and water were purchased from Honeywell (Charlotte, NC, USA), and ammonium bicarbonate, methylenediphosphonic (medronic) acid, ammonium hydroxide and formic acid were purchased from Sigma-Aldrich. The sodium carboxymethylcellulose (CMC) used for preparing zebrafish for DESI imaging was purchased from Sigma-Aldrich. Hydroxychloroquine sulfate was pharmaceutical grade and purchased from Sigma-Aldrich, ^13^C_6_-glucose was purchased from Cambridge Isotopes (Tewksbury, MA, USA), and penicillin-streptomycin was purchased from Life Technologies (Carlsbad, CA, USA). The labeled HCQ-D_4_ internal standard used for absolute quantitation was purchased from Cayman Chemical (Ann Arbor, MI, USA).

### Zebrafish husbandry

Use of animals in this study was conducted in accordance with the Washington University Institutional Animal Care and Use Committee regulations. All zebrafish were in-bred AB strain (sjA) and were reared by using standard practices (Nechiporuk et al., 1999; Westerfield, 2000). Fish were maintained at a temperature of 28±1°C under a 14:10 hr light:dark circadian cycle. All experiments were performed by using adult, age-matched, randomly selected male and female zebrafish, except for DESI imaging. DESI-imaging experiments utilized only males for anatomical consistency in slicing and spatial distribution. Prior to use in experiments, fish were fasted for 24 h to clear the intestine. Treatments were conducted in washed, autoclaved beakers with conditioned facility water, in which the fish were allowed to swim freely. All experiments were conducted at a consistent density, no greater than one fish per 100 ml water. During all treatments, beakers were maintained under the same pre-experimental conditions (temperature, light cycle) by using a heated water bath and timed lighting. Sample sizes for individual experiments are provided in their corresponding figure legends.

Zebrafish were anesthetized and euthanized by using gradual cooling to minimize perturbations to blood glucose due to chemical anesthesia (Eames et al., 2010; Naser et al., 2021). For anesthesia, zebrafish were placed in a beaker with 100 ml facility water at 17°C. The beaker was placed in a shallow ice bath and the temperature was lowered over the course of approximately 5 min to 12°C, when stage 3, phase 2 anesthesia was reached. For euthanasia, zebrafish were placed in a beaker with 100 ml facility water and ice for at least 10 min. Euthanasia was deemed complete 1 min after the cessation of opercular movements.

### Drug-treatment experiments

For initial survival-curve data, adult zebrafish (*n*=3 per group) were placed in treated facility water containing 2% penicillin/streptomycin as well as concentrations of HCQ ranging from 1 µM to 10 mM. Survival was documented every 24 h, and non-surviving fish were immediately removed. Once drug tolerance was gauged with the pilot study, the experiment was repeated and expanded to include larger sample sizes (*n*=10 animals per group) and more intermediate concentrations.

For initial pharmacokinetic plots, adult zebrafish were placed in treated facility water containing 2% penicillin/streptomycin as well as 1 mM HCQ for up to 48 h. Fish were euthanized, their organs harvested, and HCQ levels analyzed at 0, 12, 24 and 48 h of exposure. For all further LC/MS experiments, adult zebrafish were placed in treated facility water containing 2% penicillin/streptomycin (to prevent bacterial growth). Drug-treated groups were administered 1 mM HCQ for 24 h.

For all experiments, *n*-values are listed in the figure legends. For some experiments, tissues have different *n*-values. This difference in *n*-values between tissues is because we were unable to successfully harvest each tissue from all animals. We also point out that not every experiment used the same number of animals. This is because some fish died during experiments and were not included in the study.

### Serum and tissue harvest and extraction

Serum and tissues were harvested and extracted from zebrafish as previously described (Naser et al., 2021). Briefly, fish were euthanized by using gradual cooling and thoroughly dried before caudal fin amputation for blood collection. Fish were immediately transferred (wound side down) to a blood-collection device consisting of one 1.5 ml microcentrifuge tube (holding tube) with a small (∼0.5 mm) hole in the bottom attached to a 0.5 ml microcentrifuge tube (collection tube). Zebrafish were centrifuged at 40 ***g*** for 1 min at 15°C. The collected blood was placed on ice for 10 min to clot. During this time, organs were dissected from the fish, placed in pre-weighed 1.5 ml microcentrifuge tubes, and snap frozen in liquid nitrogen. After 10 min, the blood was centrifuged at 1600 ***g*** for 10 min at 4°C. Immediately, the serum was pipetted into a new 0.5 ml microcentrifuge tube and snap frozen in liquid nitrogen. All samples were stored at −80°C until extraction.

For metabolite extraction, tissue samples were ground to a powder in liquid nitrogen by using a disposable plastic pestle. Tissue samples were immediately weighed and a normalized volume of 40 µl of 2:2:1 methanol:acetonitrile:water per 1 mg tissue was added. Samples were vortexed, snap frozen, and stored at −80°C. At the same time, all tissue samples were extracted by using the following sequence: thawing in a room-temperature water bath, sonication at room temperature for 5 min, vortexing, snap freezing in liquid nitrogen, thawing in a room-temperature water bath, sonication at room temperature for 5 min, vortexing, and incubation for 1 h at −20°C. After incubation, samples were centrifuged at 20,000 ***g*** for 10 min at 4°C. Supernatants were transferred to LC/MS vials for analysis.

Metabolite extraction of serum samples was completed by using a different method from tissue samples. Serum samples were thawed on ice and diluted 1:15 in 2:2:1 methanol:acetonitrile:water. Samples were mixed and incubated at −20°C for 1 h. After incubation, samples were centrifuged at 20,000 ***g*** for 10 min at 4° C. Supernatants were transferred to LC/MS vials for analysis.

### Absolute quantitation experiment

To generate calibration curves, untreated control fish were carried through the organ harvesting and metabolite extraction process to generate tissue and serum samples for metabolite extraction (in 2:2:1 methanol:acetonitrile:water). From these extracts, a pool was created containing either an equal-parts mixture of all organs from all fish, or an equal-parts mixture of serum extract from all fish. The HCQ-D_4_ internal standard was added to the pooled extracts at 1 µg/ml. Using this matrix, HCQ (unlabeled solid) was dissolved and serially diluted (1:10) to produce two calibration curves, one for serum and one for organs, each consisting of five concentration points ranging from 10 ng/ml to 100 µg/ml HCQ in 100 µl extract. Each calibration curve was handled identically to its corresponding samples to be quantified (organs or serum), being carried through the same extraction process and analyzed simultaneously by LC/MS.

For absolute quantitation in treated animals, adult zebrafish were placed in 1 mM HCQ for 24 h. At the end of the experiment, fish were euthanized and organs were harvested and snap frozen. Metabolite extraction in tissues and serum was carried out by using methods identical to those described above, with the addition of 1 µg/ml HCQ-D_4_ added directly to the 2:2:1 methanol:acetonitrile:water solvent as an internal standard.

### DESI-based imaging

MS imaging experiments utilized three treated (1.5 mM HCQ for 24 h) and three non-treated control male zebrafish. Immediately after treatment, fish were euthanized, a portion of the tail was excised and each fish was centrifuged for 1 min at 40 ***g*** for blood collection, as described above. Fish were transported on dry ice, embedded in 5% sodium CMC in water as a stabilizing matrix, and stored at −80°C. Fish were sectioned at 20 µm thickness by using a CM 1860 Cryostat (Leica Biosystems). The CMC matrix was injected into the swim bladders once exposed by the cryostat and allowed to freeze completely to mechanically stabilize tissue. Sections were thaw-mounted to Superfrost Plus Glass Slides (Thermo Fisher Scientific). Slides were cleaned with methanol immediately before use. Collected sections were dried under vacuum and stored at −80°C. Sections used for imaging were thawed under vacuum to limit tissue rehydration.

DESI imaging was performed by using a Synapt XS quadrupole time-of-flight mass spectrometer with a traveling wave ion mobility cell and a DESI-MSI source (Waters Corporation, Milford, MA, USA). Imaging was performed by using 4.0 kV capillary voltage in negative polarity for Resolution Mode. DESI spray used 95:5 methanol:water with 0.1% formic acid and 200 pg/µl leucine enkephalin (Waters Corporation) at a 2.5 µl/min flow rate, 70° incident angle and −7° collection angle. Images were collected at a 25 µm/s raster rate for 150 µm×150 µm pixel size. At least two serial sections were imaged for each fish.

The total ion current (TIC) was collected for each section of the fish, and relevant mass-to-charge ratios (*m/z*) were extracted. For non-normalized images, the values are given as relative intensities. For normalized images (Fig. S1C), the intensities of the extracted *m/z* values for each pixel are divided by the TIC for the same pixel.

### LC/MS analysis

All LC/MS analyses in this work were conducted by using a Q Exactive Plus mass spectrometer coupled to a Dionex Ultimate UHPLC system (Thermo Fisher Scientific). Chromatographic separation was performed by hydrophilic interaction liquid chromatography (HILIC) on the iHILIC-(P) Classic column (100 mm×2.1 mm, 5 µm) (HILICON, Umeå, Sweden) preceded by a corresponding iHILIC-(P) Classic guard column (20 mm×2.1 mm, 5 µm). Mobile-phase solvent A was 95:5 water:acetonitrile (ACN) with 20 mM ammonium bicarbonate, 0.1% ammonium hydroxide and 2.5 µM medronic acid, and solvent B was 95:5 ACN:water with 2.5 µM medronic acid. Samples were separated at 0.25 ml/min over a 14 min analysis frame with a 12 min linear gradient, as follows: 0-1 min, held at 90% solvent B; 1-13 min, ramped 90% to 30% solvent B; 13-14 min, held at 30% solvent B. Following scanning, the column was flushed at 90% solvent B for 5 min at an increased flow rate (0.4 ml/min) to ensure equilibration before the next injection, prior to which the flow rate was returned to 0.25 ml/min. The injection volume was 3 µl. Before and after each injection, the needle was washed with 100 µl 1:1 water:ACN. Analysis sequences were organized with samples in a randomized order consisting of blocks of ten separated by a solvent blank (2:2:1 methanol:acetonitrile:water) and a pooled quality control (QC) sample . Three solvent blanks were run both at the beginning and end of the sequence. For absolute quantitation, calibration curves were analyzed in triplicate with their corresponding quantitated samples. All analyses, except for HCQ absolute quantitation, were performed in full-scan mode with a scan range of 65-950 *m/z*, 70 K resolution (140 K for isotope labeling), maximum ion time of 100 ms and automatic gain control (AGC) target of 10^6^. Absolute quantitation was performed by using a targeted single-ion monitoring method selecting for HCQ and its labeled internal standard (HCQ-D_4_) with an isolation window of 2 *m/z*, 35 K resolution, maximum ion time of 75 ms and AGC target 10^5^. MS source settings were identical for all analyses, as follows: spray voltage, −3 kV; sheath gas flow rate, 50; auxiliary gas flow rate, 10; sweep gas flow rate, 1; S-lens RF level, 30%; capillary temperature, 300°C; and auxiliary gas heater temperature, 350°C.

Analytical performance and reproducibility for untargeted and isotope-labeling experiments were evaluated by examining peak area ratios of a panel of known metabolites in the pooled QC sample, all of which showed relative standard deviations (RSDs) <15% across the analysis, indicating high reproducibility. For absolute quantitation, the HCQ-D_4_ internal standard peak area was used for QC, which showed intra-group RSD<15% for all organs and serum. Performance of the calibration curves for organs and serum (triplicate data for each combined into a single curve for quantitation) was assessed by overall linearity, accuracy and reproducibility of each calibrator point. Both calibration curves were found to show excellent linearity (R^2^>0.999), accuracy and reproducibility (RSD<10%).

### Kidney studies

Kidney tissue was harvested from adult zebrafish in separate experiments. Metabolites were extracted and analyzed with LC/MS by using the same methods as detailed above. The samples were profiled to identify global changes in metabolism, but absolute quantitation of HCQ in kidney was not performed. The annotation of kidney as having high accumulation of HCQ in Fig. 3B is based on relative quantitation as assessed by DESI imaging. We also note that, for the treatment group, fish were placed in 1 mM HCQ for 24 h prior to harvesting kidney tissue. We assumed that kidney HCQ levels were at steady state by 24 h. Although this was not formally measured, every other tissue we examined was at steady state by 24 h (Fig. 1B).

### Isotope-tracer experiments

For isotope tracing, 10 mM ^13^C_6_-glucose was added to both untreated control and 1 mM HCQ-treated conditions. Throughout the experiment, fish were allowed to swim freely. After 24 h, both HCQ levels and isotope enrichment were at steady state in the fish, and this marked the endpoint of the experiment. Fish were then euthanized, their organs harvested, and samples analyzed by LC/MS.

### Data processing, statistical analysis and metabolite identifications

Data processing for this study was performed by using several software, depending on the specific application. For analysis of the quantitative data, including peak picking for HCQ or HCQ-D_4_ and calculation peak area ratios in samples and calibration curves, Quan Browser (Xcalibur Workstation, Thermo Fisher Scientific) was used with an optimized processing method. Data processing for isotope-tracing experiments was performed by using Skyline (peak picking) and AccuCor (^13^C natural-abundance correction) (MacLean et al., 2010; Su et al., 2017). Data processing of the untargeted metabolomics data was accomplished by using Compound Discoverer Version 3 Series (Thermo Fisher Scientific). Statistical significance was determined by using a two-tailed, unpaired Student's *t*-test, except for one-way ANOVA analysis (as labeled in figure legend) using GraphPad Prism (version 9) with Tukey's method at 95% confidence. Linear coefficients were calculated with GraphPad Prism (version 9) by using a two-tailed Pearson correlation at 95% confidence. All metabolites assigned a biochemical name were identified at level 1 confidence in accordance with the Metabolomics Standards Initiative by matching experimental data from zebrafish to data from authentic reference standards that we generated in our laboratory by using the specific methods applied in this work (Sumner et al., 2007; Stancliffe et al., 2021). Identifications were supported by matching accurate mass measurements (≤10 ppm) and retention times for LC/MS experiments or ion-mobility drift times for DESI experiments. DESI-imaging data were processed by using High Definition Imaging (Waters Corporation) to perform peak picking and lockmass correction against leucine enkephalin. Images were prepared from processed files by using a custom Python script and ImageJ. Outliers were calculated by the Grubbs’ Test with α at 0.05. Metabolomics data are available in Table S2.

## Supplementary Material

Supplementary information
