# Supplementary material for: A multidimensional metabolomics workflow to image biodistribution and evaluate pharmacodynamics in adult zebrafish
Source: Dis Model Mech. 2022 Aug 16;15(8):dmm049550. doi: 10.1242/dmm.049550 (PMC9411795; doi:10.1242/dmm.049550)
Supplement: Supplementary information [file dmm-15-049550-s1.pdf]

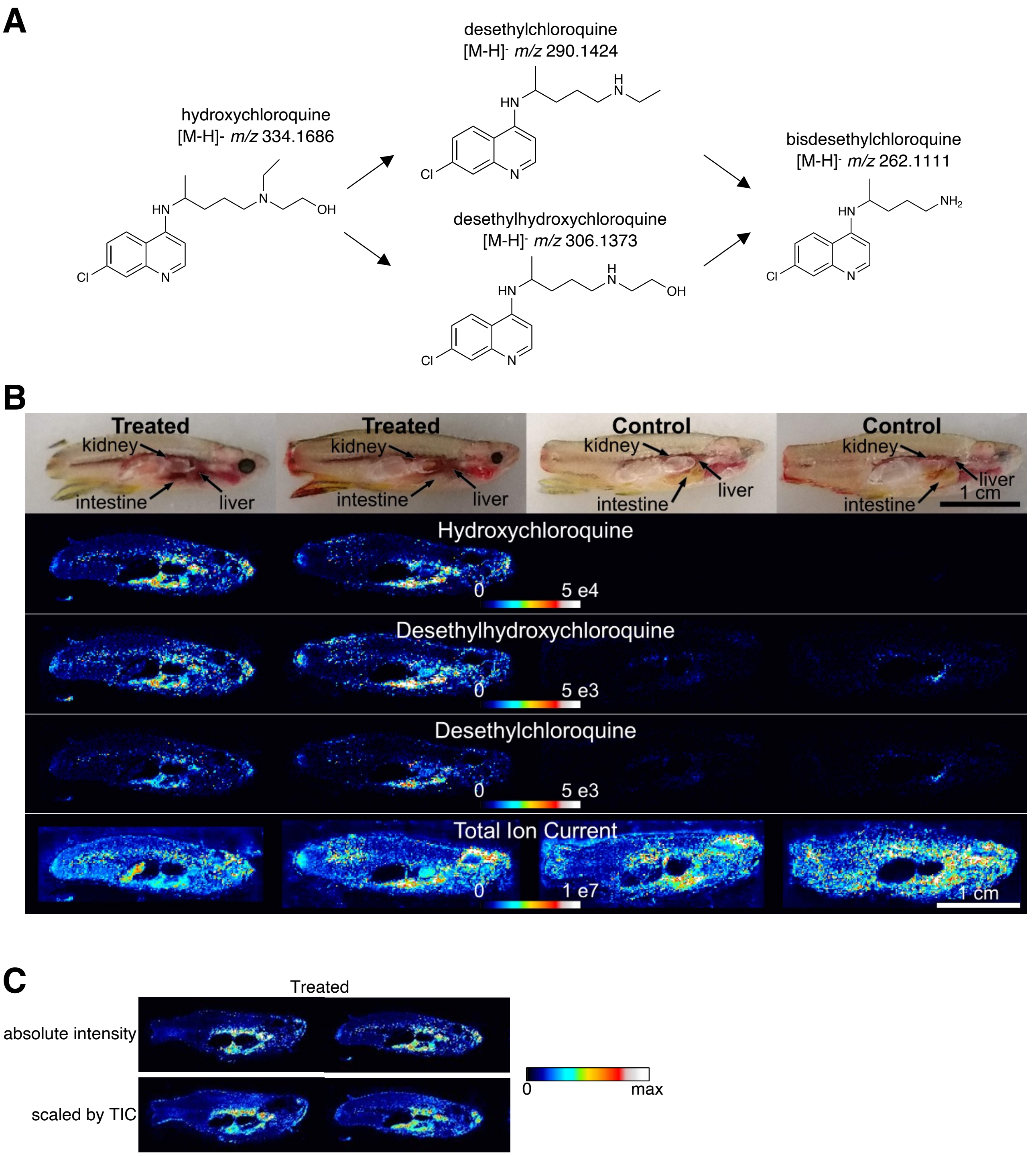

Fig. S1. HCQ metabolites and their localization with DESI-MS imaging.

A) Schematic of HCQ metabolism.

B) DESI imaging of replicate HCQ-treated and control zebrafish. Annotated reference photographs for representative collected sections. HCQ and its metabolites desethylhydroxychloroquine and desethylchloroquine are detected in high abundance (compared to control fish), with enrichment in intestine, liver, and kidney. Comparable intensities among total ion current images confirm stable DESI performance.

C) DESI imaging of HCQ-treated zebrafish. The top image displays absolute signal intensity. The bottom image displays absolute signal intensity scaled by the TIC.

Disease Models & Mechanisms • Supplementary information

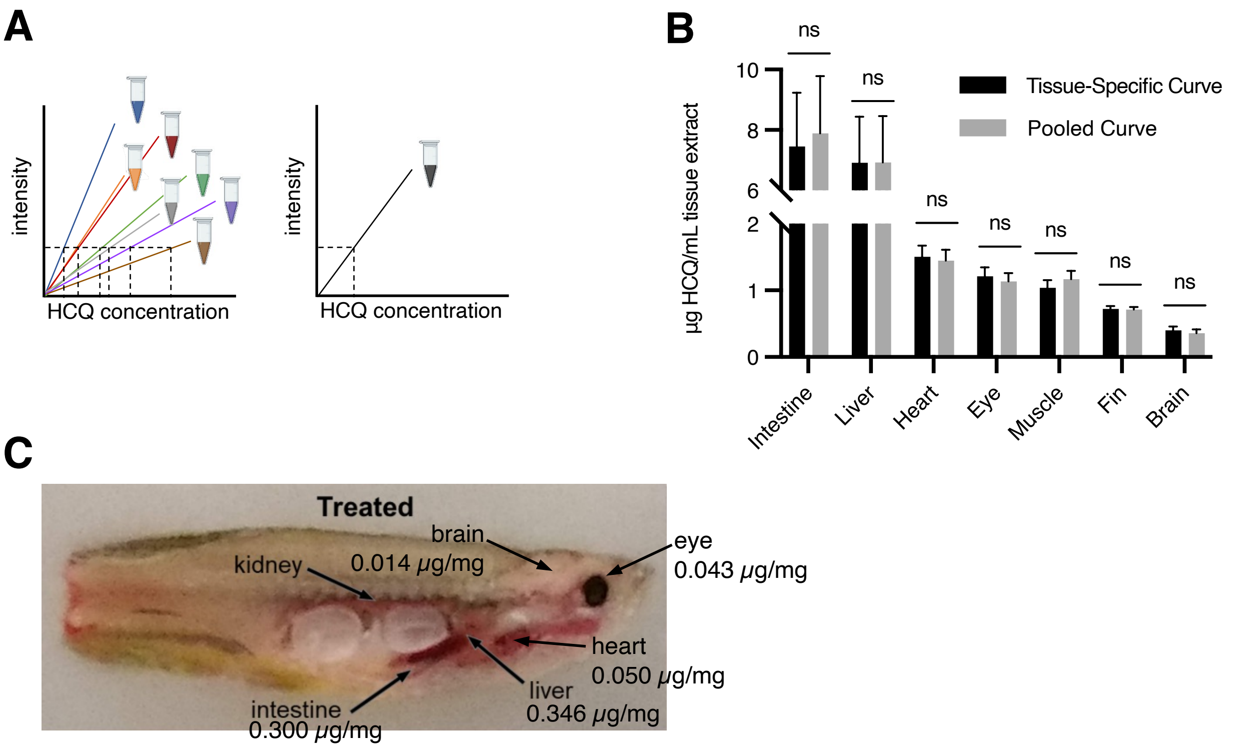

Fig. S2. Use of a calibration curve from pooled samples for absolute quantitation of HCQ.

A) Schematic to show utility of a single calibration curve from pooled samples. In place of running calibration curves for individual organ types, a pooled sample was used to determine HCQ concentration in all organs tested. A calibration curve from the mixed sample accurately accounts for the matrix effects of different organs.

B) Absolute quantitation of HCQ in a tissue-specific curve and a pooled calibration curve. Values are mean ±SEM of replicate injections; n = 5-8 fish. For intestine n = 7; for liver, heart, eye, muscle, fin, and brain n = 8.

C) Annotated reference photograph to demonstrate HCQ localization from LC/MS data as it relates to signal intensity from DESI analysis. Statistical significance was evaluated with a two-tailed, unpaired t test and annotated as follows: ns = not significant.

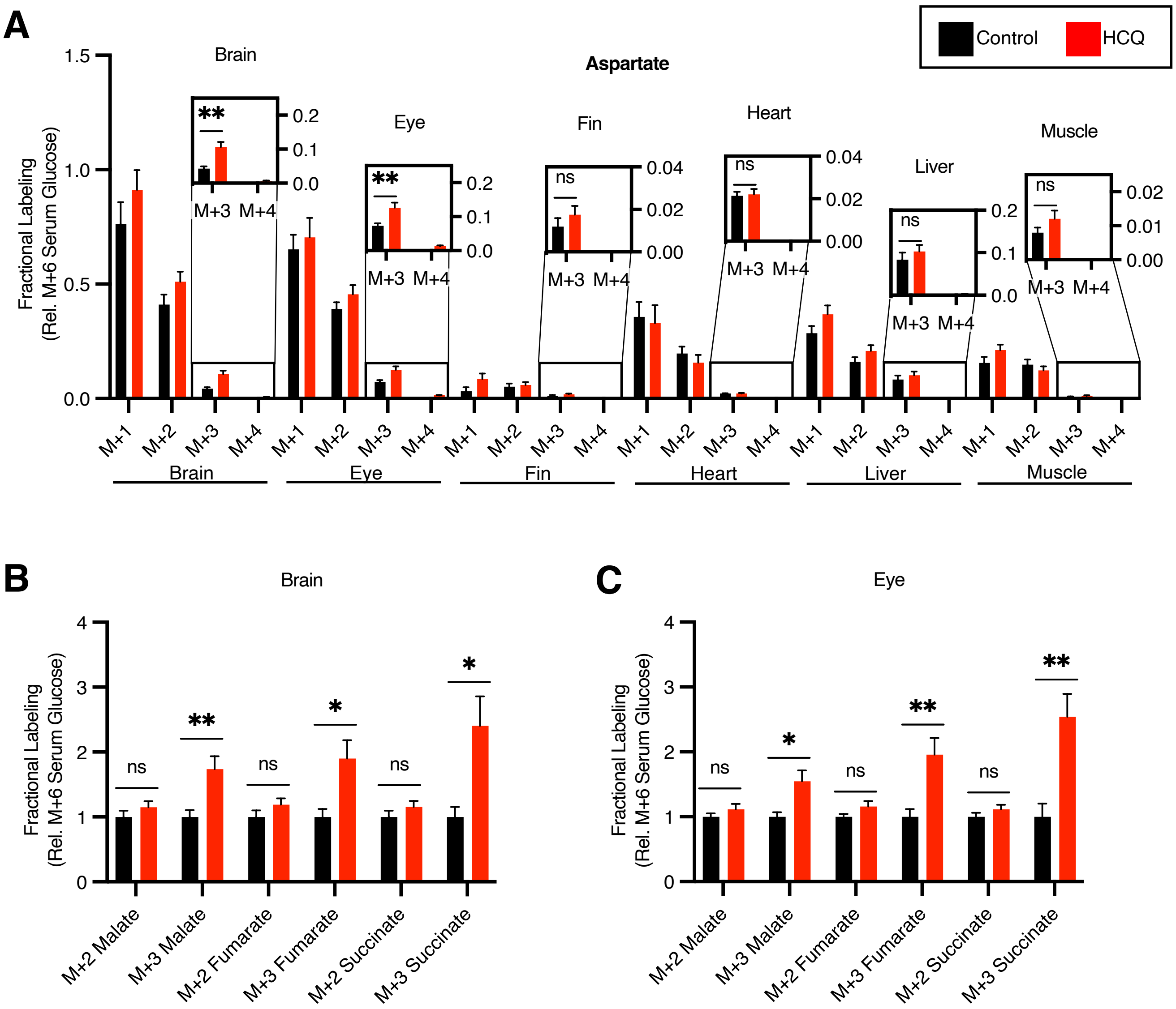

Fig. S3. Stable-isotope tracing to probe perturbations to TCA cycle metabolism with HCQ treatment

A) Expanded graph from Figure 4D of fractional labeling of aspartate from 13C6-glucose in HCQ and control zebrafish; n = 6-9 fish per group. For control, brain n = 6; eye, fin, heart, liver, and muscle n = 8. For HCQ, heart and liver n = 8; brain, eye, fin, muscle n = 9.

B) Fractional labeling (relative to serum M+6 glucose) showed increased M+3 malate, fumarate, and succinate, relative to M+2 isotopologues, in brain tissue of HCQ-treated (ZF); n = 6-9 per group. For control brain n = 6. For HCQ brain n = 9.

C) Fractional labeling (relative to serum M+6 glucose) showed increased M+3 malate, fumarate, and succinate, relative to M+2 isotopologues, in eye tissue of HCQ-treated zebrafish (ZF); n = 6-9 per group. For control eye n = 8. For HCQ eye n = 9.

Statistical significance was evaluated with a two-tailed unpaired t test and annotated as follows: *p<0.05, **p<0.01, ns = not significant.

Disease Models & Mechanisms • Supplementary information

Table S1. Calibration-curve equations. All calibration curves were run in triplicate and a single calibration curve was generated by using QuanBrowser. Pearson *r* values were calculated by using GraphPad Prism, with the following parameters: two tailed, 95% confidence. Calibration curves were based on the concentration of hydroxychloroquine sulfate. Reported concentrations of HCQ were numerically corrected to account for the mass of sulfate and for reported values in figures. Outliers were removed from these calibration equations.

Sample Type	Calibration Curve Equation	R^2	Pearson r
Brain	Y = -0.000201502+0.131123*X	0.9995	0.9994
Eye	Y = -0.000433154+0.136172*X	0.9998	0.9999
Fin	Y = 0.000992346+0.138859*X	0.9996	0.9997
Heart	Y = 0.000565904+0.141306*X	0.9996	0.9998
Intestine	Y = -0.00114644+0.139627*X	0.9998	0.9999
Liver	Y = -0.00087811+0.143639*X	0.9994	0.9998
Muscle	Y = 0.0347205+0.160256*X	0.9997	0.9997
Pooled	Y = 0.00828276+0.139247*X	0.9991	0.9986

Table S2. Metabolomics Data

Click here to download Table S2

Disease Models & Mechanisms • Supplementary information
